# Reliability and validity of the Korean standard pattern identification for stroke (K-SPI-Stroke) questionnaire

**DOI:** 10.1186/1472-6882-12-55

**Published:** 2012-04-26

**Authors:** Byoung-Kab Kang, Tae-Yong Park, Ju Ah Lee, Tae-Woong Moon, Mi Mi Ko, Jiae Choi, Myeong Soo Lee

**Affiliations:** 1 Medical Research Division, Korea Institute of Oriental Medicine, Daejeon, Republic of Korea

**Keywords:** Reliability, Validity, Pattern identification, Stroke

## Abstract

**Background:**

The present study was conducted to examine the reliability and validity of the ‘Korean Standard Pattern Identification for Stroke (K-SPI-Stroke)’, which was developed and evaluated within the context of traditional Korean medicine (TKM).

**Methods:**

Between September 2006 and December 2010, 2,905 patients from 11 Korean medical hospitals were asked to complete the K-SPI-Stroke questionnaire as a part of project ' Fundamental study for the standardization and objectification of pattern identification in traditional Korean medicine for stroke (SOPI-Stroke). Each patient was independently diagnosed by two TKM physicians from the same site according to one of four patterns, as suggested by the Korea Institute of Oriental Medicine: 1) a Qi deficiency pattern, 2) a Dampness-phlegm pattern, 3) a Yin deficiency pattern, or 4) a Fire-heat pattern. We estimated the internal consistency using Cronbach’s α coefficient, the discriminant validity using the means score of patterns, and the predictive validity using the classification accuracy of the K-SPI-Stroke questionnaire.

**Results:**

The K-SPI-Stroke questionnaire had satisfactory internal consistency (α = 0.700) and validity, with significant differences in the mean of scores among the four patterns. The overall classification accuracy of this questionnaire was 65.2 %.

**Conclusion:**

These results suggest that the K-SPI-Stroke questionnaire is a reliable and valid instrument for estimating the severity of the four patterns.

## Background

Stroke, the second most common cause of death in Korea, is a considerable burden on Korean society. According to an annual report of the National Health Insurance Corporation in 2009, the estimated cost to treat and manage cerebrovascular diseases totals 920 billion won (approximately 0.87 billion dollars) [1].

Historically, traditional Korean medicine (TKM), which considers- the location, cause, and nature of patients’ diseases, has been the major treatment modality in the Korean medical system for properly treating stroke patients.

TKM has a unique diagnostic system called pattern identification (PI), which distinguishes it from modern Western medicine. PI is a process of the overall analysis of clinical data using an integrative approach that addresses the etiology, pathology, and treatment method [2]. This diagnostic system is theoretically well organized and involves the classification of patients into certain categories. However, other medical systems did not recognize the pattern classifications of TKM as a diagnostic system by TKM doctors, which prompted these doctors to study whether they could make the same diagnosis in identical patients (reliability) and whether their diagnoses were accurate (validity). Considering the importance of PI in determining treatment regimens, an accurate questionnaire is warranted.

In the last five years, many TKM doctors have studied the diagnostic validity and reliability of TKM for various health conditions [2-6]. Lee et al. [7] released the Korean Standard Pattern Identification for Stroke (K-SPI-Stroke) in 2011, which included the sign and symptoms used by TKM doctors to diagnose patterns of stroke in Korean patients. We evaluated the reliability and validity of the K-SPI-Stroke.

## Methods

### Study subjects and data collection

This study was a community-based multi-center trial. Stroke patients were admitted to 11 TKM hospitals. We enrolled stroke patients within 30 days of the onset of their symptoms as confirmed by imaging diagnosis, such as computerized tomography (CT) or magnetic resonance imaging (MRI). We excluded traumatic strokes, such as subarachnoid, subdural, and epidural hemorrhages. Moreover, we excluded patients who exhibited a blood stasis pattern. Originally, blood stasis was one of the possible patterns, but the number of patterns was changed from four patterns to five pattern in a previous study [7] because blood stasis was dropped due to a small sample size. Informed consent was received from all of the subjects, and PI was performed using the 44 signs and symptoms related to the four patterns. On the same day, to minimize differences between the two diagnoses, patients were independently diagnosed with one of the four patterns using PI by two physicians. The physicians had at least three years of clinical experience with stroke after finishing their regular (six years) education in TKM. The physicians took regular training courses on standard operating procedures (SOP) twice a year. The number of physicians at each site ranged from 2 to 25, and a total of 105 physicians were included in this study. On the basis of the diagnostic results from the physicians, the patients were classified to have an identical pattern when the two physicians’ decisions were in agreement. Over 40 months, 2,905 patients participated in this study (from September 2006 to December 2010). This study was approved by the Institutional Review Boards of the Korea Institute of Oriental Medicine (KIOM) and each of the participating TKM hospitals.

### Pattern identification questionnaire

The K-SPI-Stroke questionnaire was developed in a previous study via a multistage process [8]. First, the signs and symptoms included in the K-SPI-Stroke were selected from the TKM literature, textbooks, and clinical papers by an expert committee organized by the KIOM. Second, a K-SPI-Stroke prototype featuring various signs and symptoms was applied to test the data from recruited patients. The K-SPI-Stroke was then reexamined by a committee of experts and adjusted to maximize its validity. Consequently, the final version of the K-SPI-Stroke included 44 sign and symptom entries (11 Qi deficiency pattern items, 7 Dampness-phlegm pattern items, 7 Yin deficiency pattern items and 19 Fire-heat pattern items) [7,9,10]. The severity for each item is graded on the following scale: 1 = not significant, 2 = significant and 3 = very significant. To convert this scale to a dichotomous scoring system, 0 points were assigned for a grade score of 1, and 1 point was given for each item receiving a graded score of 2 or 3. The dichotomous scores of each item were summed, and the K-SPI-Stroke score (Qi deficiency pattern score: QDP score, Dampness-phlegm pattern score: DPP score, Yin deficiency pattern score: YDP score, and Fire-heat pattern score: FHP score) was calculated.

### Statistical methods

The internal consistency of the questionnaire was assessed using Cronbach’s α coefficient. It has been suggested that values of α > 0.5 are acceptable, although ideally the scores should be >0.7 [11,12]. The K-SPI-Stroke score of each pattern was calculated by summing all of the relevant item scores for each pattern category. As an indication of the discriminant validity, the mean score of the patients’ diagnosed pattern should be significantly higher than the other nondiagnosed patterns, indicating the scores’ ability to differentiate among the patterns. The underlying hypothesis was that stroke patients would report higher scores for their diagnosed pattern and thus report lower scores for the other nondiagnosed patterns. To test the discriminant validity, which test whether measures that are supposed to be unrelated are in fact unrelated [13], we used a one-way analysis of variance (ANOVA to compare the mean scores of the four patterns. Posteriori comparisons were performed using Scheffé’s test. To evaluate the predictive validity, we used a classification accuracy test with a discriminant linear function for the four patterns. Statistical significance was achieved when a P value was less than 0.05. All analyses were performed with SAS version 9.1.3 (SAS Institute, Cary, NC).

## Results

### Demographic characteristics of patients with stroke

The mean age of the 2,905 subjects was 66.98 (standard deviation, 11.55) years. The numbers of male and female were 1,530 (52.67%) and 1,375 (47.33%), respectively. Approximately 65% of the patients had histories of drinking and smoking. Patients with diabetes and hyperlipidemia accounted for 26.73% and 12.63% of the population, respectively. The frequency of stroke patients with hypertension (60.54%) was higher than the frequency of patients with diabetes or hyperlipidemia. Other variables are also presented in Table 1.

**Table 1:** Demographic characteristics of patients with stroke

**Variables**	**mean (SD), no. (%)**	
Age (yrs), mean(SD)		66.98 (11.55)	
Gender, no. (%)			
Male	1530		52.67 %
Female	1375		47.33 %
Drinking history, no. (%)			
No	1041		35.91 %
Yes	1858		64.09 %
Smoking history, no. (%)			
No	862		29.74 %
Yes	2036		70.26 %
Weight (kg), mean(SD)		57.05 (9.62)	
Height (cm), mean(SD)		157.92 (8.50)	
Marriage status, no. (%)			
Single	62		2.16 %
Married	2049		71.37 %
Divorce	62		2.16 %
Widowed	698		24.31 %
Diabetes, no. (%)			
No	2113		73.27 %
Yes	771		26.73 %
Hyperlipidemia, no. (%)			
No	2498		87.37 %
Yes	361		12.63 %
Hypertension, no. (%)			
No	1140		39.46 %
Yes	1749		60.54 %
Anticoagulant treatment, no. (%)			
No	1913		67.86 %
Yes	906		32.14 %
NIHSS score, mean(SD)		4.32 (3.78)	

*SD* standard deviation.

### Reliability analysis

Cronbach’s α coefficient was computed to test the internal consistency of the 44 items on the K-SPI-Stroke questionnaire. Cronbach’s α was 0.70 for the 44 signs and symptoms, indicating that the K-SPI-Stroke exhibited good internal consistency and the alpha values for the patterns ranged from 0.42 to 0.67 (Table 2).

**Table 2:** Internal consistency (Cronbach’s coefficient α) total and within each pattern and mean scores for each item of patterns

**Pattern**	**Symptoms and Signs**	**Alpha if item deleted**	**Qi deficiency**	**Dampness-phlegm**	**Yin deficiency**	**Fire-heat**
**Total**		**α = 0.700**				
**Qi deficiency**	**α = 0.674**					
Drowsiness, likes to lie down		0.696	1.18 (0.97)	0.99 (0.75)	1.03 (0.84)	0.93 (0.75)
Feels powerless and lazy		0.693	1.85 (0.73)	1.46 (0.6)	1.61 (0.65)	1.38 (0.59)
Looks powerless and lazy		0.693	1.94 (0.66)	1.44 (0.57)	1.62 (0.61)	1.35 (0.53)
Pale complexion		0.694	1 (1.05)	0.29 (0.61)	0.39 (0.7)	0.26 (0.54)
Reluctance to speak		0.690	1.58 (0.68)	1.25 (0.5)	1.34 (0.59)	1.21 (0.5)
Pale tongue		0.694	0.81 (1.02)	0.74 (0.96)	0.43 (0.78)	0.4 (0.75)
Teeth-marked tongue		0.695	0.54 (0.88)	0.5 (0.8)	0.37 (0.66)	0.38 (0.67)
Slow pulse		0.694	0.77 (0.98)	0.54 (0.82)	0.45 (0.71)	0.37 (0.67)
Weak pulse		0.694	1.35 (1.04)	0.6 (0.87)	0.92 (0.97)	0.44 (0.74)
Fine pulse		0.702	1.42 (0.6)	1.08 (0.36)	1.38 (0.57)	1.06 (0.35)
Reversed cold in the extremities		0.697	1.11 (0.57)	1.02 (0.47)	1.05 (0.49)	1 (0.39)
**Dampness-phlegm α = 0.424**						
Sallow complexion		0.705	0.58 (0.88)	1.2 (0.94)	0.6 (0.84)	0.45 (0.75)
Dark inferior palpebral		0.705	1.17 (0.39)	1.22 (0.43)	1.14 (0.4)	1.11 (0.37)
Dizziness with nausea		0.688	1.12 (0.42)	1.19 (0.5)	1.16 (0.47)	1.13 (0.44)
White fur on tongue		0.703	1.28 (1)	1.36 (0.99)	0.92 (0.98)	0.91 (1.02)
Enlarged tongue		0.691	0.41 (0.76)	0.56 (0.83)	0.39 (0.67)	0.42 (0.72)
Slippery pulse		0.706	1.2 (0.51)	1.75 (0.55)	1.24 (0.5)	1.4 (0.58)
Heavy		0.693	0.34 (0.72)	0.82 (1.02)	0.44 (0.74)	0.62 (0.91)
**Yin deficiency α = 0.525**						
Pale face and red zygomatic-site		0.686	0.19 (0.52)	0.25 (0.58)	0.8 (0.95)	0.34 (0.69)
Dry mouth		0.690	1.54 (0.67)	1.45 (0.61)	1.78 (0.72)	1.6 (0.73)
Dry fur on tongue		0.689	0.47 (0.77)	0.56 (0.76)	0.99 (1.01)	0.84 (0.95)
Bare and red tongue like a mirror		0.683	0.16 (0.46)	0.17 (0.4)	0.49 (0.83)	0.25 (0.51)
Night sweating		0.696	1.17 (0.46)	1.16 (0.43)	1.3 (0.56)	1.21 (0.48)
Tidal fever		0.696	1.05 (0.31)	1.05 (0.28)	1.19 (0.47)	1.1 (0.4)
Thin		0.690	0.37 (0.77)	0.28 (0.59)	0.66 (0.96)	0.48 (0.79)
**Fire-heat α = 0.599**						
Vexation and insomnia		0.689	0.42 (0.65)	0.47 (0.69)	0.65 (0.84)	0.66 (0.88)
Reddened complexion		0.700	0.31 (0.7)	0.48 (0.82)	0.6 (0.91)	1.43 (1.02)
Headache like flush		0.696	0.33 (0.56)	0.28 (0.51)	0.33 (0.58)	0.33 (0.61)
Blood-shot eyes		0.713	1.6 (0.6)	1.49 (0.55)	1.5 (0.56)	1.54 (0.6)
Wheezing in the throat with sputum		0.715	1.08 (0.54)	1.17 (0.64)	1.08 (0.63)	1.14 (0.64)
Aphtha or tongue sore		0.694	0.94 (0.38)	0.94 (0.42)	1 (0.43)	1.03 (0.38)
Fetid mouth odor		0.693	1.07 (0.5)	1.2 (0.56)	1.11 (0.54)	1.29 (0.63)
Thirst		0.694	1.32 (0.59)	1.3 (0.55)	1.45 (0.66)	1.51 (0.73)
Red tongue		0.694	0.43 (0.81)	0.5 (0.82)	1.08 (1.04)	1.09 (1.05)
Yellow fur		0.694	0.45 (0.83)	0.7 (0.94)	0.66 (0.89)	1.07 (1.07)
Thick fur		0.691	0.68 (0.95)	1.15 (1.01)	0.72 (0.87)	1.22 (1.05)
Heat vexation in the chest		0.683	0.46 (0.7)	0.43 (0.68)	0.62 (0.82)	0.59 (0.87)
Turbid urine		0.695	1.15 (0.6)	1.05 (0.66)	1.2 (0.58)	1.13 (0.7)
Rapid pulse		0.694	0.48 (0.81)	0.76 (0.94)	1 (1.04)	1.05 (1.01)
Strong pulse		0.702	0.46 (0.8)	0.96 (0.98)	0.68 (0.91)	1.43 (1)
Surging pulse		0.702	1 (0.24)	1.04 (0.29)	1.01 (0.29)	1.19 (0.49)
Heat vexation and aversion to heat		0.700	1.37 (0.6)	1.41 (0.58)	1.51 (0.66)	1.71 (0.73)
Heat in the palms and soles		0.695	0.94 (0.39)	0.98 (0.45)	1.08 (0.53)	1.11 (0.49)
Vexing heat in the extremities		0.695	0.94 (0.4)	0.99 (0.46)	0.98 (0.43)	1.1 (0.5)
**Total**			40.01 (7.97)	40.25(8.45)	41.93 (8.61)	42.23 (8.78)

### Validity analysis

Because we expected to find that the mean scores of each pattern would differ depending on the diagnosed pattern of the patient, we assessed the discriminant validity using an ANOVA for the mean scores of the four patterns. We compared the means of the scores (FHP, DPP, QDP and YDP scores) to determine whether differences could be detected among the four patterns. The mean FHP score for the FHP patients was significantly higher than that for the DPP, QDP and YDP patients. The mean score of patients’ diagnosed pattern was significantly higher than the mean scores of the other patterns (Table 3).

**Table 3:** Discriminant validity of the four patterns

	QDP (n = 630)	DPP (n = 1,037)	YDP (n = 407)	FHP (n = 831)	*P-value*
QDP score	13.55 (3.96) ^A^	9.92 (3.44) ^B^	10.60 (3.37) ^B^	8.78 (3.20) ^C^	<.0001
DPP score	6.10 (2.13) ^B^	8.10 (2.42) ^A^	5.88 (2.32) ^C^	6.02 (2.27) ^C^	<.0001
YDP score	4.96 (1.98) ^B^	4.92 (2.00) ^B^	7.20 (2.51) ^A^	5.81 (2.39) ^B^	<.0001
FHP score	15.41 (3.8) ^C^	17.32 (4.34) ^B^	18.26 (4.18) ^B^	21.63 (4.80) ^A^	<.0001

The letters reflect the results from the one-way ANOVA within the same PI scores. Different letters represent a significant a posteriori comparison test between the groups of the PI.

*QDP* Qi deficiency pattern, *DPP* Dampness-phlegm pattern, *YDP* Yin deficiency pattern, *FHP* Fire-heat pattern.

To evaluate the predictive validity of this study, we used a discriminant analysis to classify the four patterns. The linear discriminant function, which consisted of the 44 items for the four patterns, is not shown. The predictive function that was taken into consideration for the distribution of the patterns and the individual patterns was assessed with the classification rates listed for the whole model. Table 4 shows the classification results for the four patterns. The overall accuracy of the classification of the four patterns was 65.2 %, and the individual classification accuracy for QDP, DPP, YDP and FHP was 64.13 %, 72.61 %, 41.77 %, and 68.23 %, respectively.

**Table 4:** Results using the classification of discriminant linear function

		**Classification result n (%)**				
		**QDP**	**DPP**	**YDP**	**FHP**	**Total**
**Physician’s diagnosis**	**QDP**	404 (64.13)	136 (21.59)	45 (7.14)	45 (7.14)	630 (21.68)
	**DPP**	95 (9.16)	753 (72.61)	37 (3.57)	152 (14.66)	1037 (35.70)
	**YDP**	65 (15.97)	77 (18.92)	170 (41.77)	95 (23.34)	407 (14.01)
	**FHP**	39 (4.69)	175 (21.06)	50 (6.02)	567 (68.23)	831 (28.61)
	**Total**	603 (20.76)	1141 (39.28)	302 (10.4)	859 (29.57)	2905 (100)

*QDP* Qi deficiency pattern, *DPP* Dampness-phlegm pattern, *YDP* Yin deficiency pattern, *FHP* Fire-heat pattern.

## Discussion

This study demonstrates that the K-SPI-Stroke questionnaire is a reliable and valid instrument. Reliability and validity are two important factors in designing a questionnaire. Reliability is concerned with the repeatability or reproducibility of the measurements while validity reflects the accuracy of the data and ensures that responses are a true reflection of the issues of interest [14-17]. To test the reliability of the K-SPI-Stroke questionnaire, we evaluated its internal consistency by using Cronbach’s α, which equals zero when the true score is not measured at all and when the data show only errors or noise; when Cronbach’s α equals 1.0, all of the items measure the true score alone without any error contributions.

The K-SPI-Stroke questionnaire had strong internal consistency, with a Cronbach’s α of 0.700 for the total signs and symptoms. However, each pattern was unsatisfactory and varied from 0.424 to 0.674. The poor reliability of the internal consistency in each pattern may suggest that the pattern constructs are not homogenous, or perhaps that the signs and symptoms are not appropriate measures of these constructs for stroke. It is likely that supplementing other patterns of K-SPI-Stroke with additional signs and symptoms, or eliminating poor signs and symptoms, may improve the reliability of the internal consistency for this questionnaire.

Cronbach’s α increased to a maximum of 0.715 when “wheezing in throat with sputum” was removed. Although this result implies that “wheezing in throat with sputum” does not measure stroke with the same validity as the other items, this item was not removed because this symptom had little influence on the overall internal consistency of the K-SPI-Stroke questionnaire.

In the analysis of the validity, we used two methods: scores comparison and a classification accuracy test. The mean score of the patients’ diagnosed pattern was significantly higher than the mean scores of the other patterns. In other words, the QDP, DPP, YDP and FHP patients reported the highest mean score for the QDP, DPP, YDP and FHP pattern, respectively. Thus, each score was a good reflection of the patient’s pathologic pattern. The second method was to compare the classification results with the physicians’ diagnoses to show the classification accuracy. The overall classification accuracy of the four patterns was 65.2 %, and the classification accuracy of the QDP, DPP, YDP and FHP was 64.13 %, 72.61 %, 41.77 %, and 68.23 %, respectively.

The YDP score was significantly higher than the scores of the other patterns (Table 2). However, the classification accuracy of the YDP (41.77 %) was lower than that of the other patterns (Table 3). This result indicates that some of the YDP items may not have accurately reflected the YDP pattern. In order words, the K-SPI-Stroke questionnaire does not discriminate YDP among the four patterns because in TKM, YDP simultaneously includes a Heat element of the FHP and a deficiency element of the QDP.

## Conclusion

A 44 items for K-SPI-Stroke was developed, which included 33 items related to signs and 11 items related to symptoms of stroke. The developed K-SPI-Stroke questionnaire had satisfactory reliability (α = 0.700), predictive validity (classification accuracy of 65.2 %) and discriminant validity with significant differences in the means of scores among the patterns. Further studies are warranted to overcome the predictive validity limitation.

## Competing interests

The authors declare that they have no competing interests.

## Authors’ contributions

BKK, TYP and MSL conceived and participated in the study design and coordination and helped to draft the manuscript. JAL, TWM, MMK, and JC participated in the study design and helped to draft the manuscript. All of the authors read and approved the final manuscript.

## Pre-publication history

The pre-publication history for this paper can be accessed here:

http://www.biomedcentral.com/1472-6882/12/55/prepub
